# Crosstalk between Oxidative Stress and Exosomes

**DOI:** 10.1155/2022/3553617

**Published:** 2022-08-30

**Authors:** Wenjun Zhang, Rong Liu, Yuhua Chen, Minghua Wang, Juan Du

**Affiliations:** ^1^School of Basic Medical Sciences, Anhui Medical University, 81 Meishan Road, Hefei, Anhui 230032, China; ^2^The Second Affiliated Hospital, The Chinese University of Hong Kong, Shenzhen, Guangdong 518172, China; ^3^Ciechanover Institute of Precision and Regenerative Medicine, School of Medicine, The Chinese University of Hong Kong, Shenzhen, Guangdong 518172, China

## Abstract

Mammals have several organs comprising various cells with different functions. Furthermore, eukaryotic cells are compartmentalized into functionally distinct organelles. Thus, for good organismal health, exosomes, which play an important role in cell-to-cell communication, interact closely with oxidative stress. Oxidative stress, which is recognized as a type of intracellular second signal, is aggravated by reactive species. As a subtype of reactive species, reactive oxygen species (ROS) can be produced on the extracellular face of the plasma membrane by NADPH oxidases, via the mitochondrial electron transport chain, in peroxisomes, and in the lumen of the endoplasmic reticulum. The scavenging of ROS is mainly dependent on peroxiredoxins, including GSH peroxidases, peroxiredoxins 3 and 5, and thioredoxin reductase. Intracellular ROS increase the number of intracellular multivesicular bodies (MVBs) by restraining their degradation in lysosomes, thereby enhancing the release of exosomes under the synergy of the depletion of exofacial GSH, which can be regulated by oxidative stress. In contrast, higher ROS levels can decrease the yield of exosomes by activating cellular autophagy to degrade MVBs. Moreover, exosomes can transfer the characteristics of parent cells to recipient cells. Here, we review the interaction between oxidative stress and exosomes in the hope of providing insights into their interplay.

## 1. Introduction

Oxidative stress is a physiological or pathological oxidative condition resulting from an imbalance between prooxidants and antioxidants or, in other words, “a disruption of redox signaling and control” as defended by Jones [1]. Oxidative stress plays several roles in regulating biological processes, including immune responses, cellular proliferation, steroidogenesis, development, ageing, thermogenesis, and cognition. However, excessive oxidant challenge leads to oxidative damage, further resulting in insulin resistance, cardiovascular disorders, and DNA damage [1–5]. The prooxidants include reactive oxygen species (ROS), reactive nitrogen species, reactive sulfur species, reactive carbonyl species, and reactive selenium species [6]. ROS are the most frequently studied subclass [7–9]. In this review, we focus on oxidative stress caused by ROS. In the studies we cite, oxidative stress was evaluated by determination of ROS levels, the levels of antioxidative enzymes, the oxidative products of biomolecules, and the levels of certain antioxidative transcription factors [6, 10]. Depending on the degree of imbalance between prooxidants and antioxidants, oxidative stress is classified as oxidative damage or oxidative eustress [6].

Extracellular vesicles are extracellular structures released by all cell types. Extracellular vesicles are delimited by a lipid bilayer and cannot undergo replication [11]. Extracellular vesicles generally include exosomes and microvesicles. Exosomes are derived from the intraluminal vesicles of multivesicular bodies generated through the endosomal pathway, with sizes ranging from 50 nm to 150 nm in diameter. Microvesicles, with a typical size range from 50 nm to 1000 nm in diameter, are generated by outward budding of the plasma membrane and subsequent microvesicle release [11–13]. However, most techniques to separate and concentrate extracellular vesicles, including differential ultracentrifugation, precipitation, density gradient separation, and size exclusion chromatography, are dependent on differences in the size or density of different subtypes of extracellular vesicles. As a result, it is difficult to completely separate exosomes from microvesicles. For the sake of rigor, many researchers use “small extracellular vesicles” interchangeably with “exosomes” to study exosomes [11]. We selected and used the nomenclature exosomes because we describe the mechanism by which oxidative stress enhances the release of exosomes in this review. Exosomes encapsulate various proteins, lipids, and nucleic acids and carry transmembrane proteins, all of which have functions in cell-to-cell communication. The precise functions of exosomes in recipient cells depend on the exosomal origin [14]. A multitude of studies have reported the functions of exosomes and related mechanisms. Below, due to space limitations, we briefly introduce two recent reports as examples. Wang et al. constructed chimeric cells by introducing nuclei from tumor cells into activated M1-like macrophages and showed that exosomes derived from chimeric cells can accumulate selectively in both lymph nodes and diverse xenograft tumors in mice, with the effect of activating T cells and ameliorating immunosuppression [15]. In addition, exosomes derived from mesenchymal stem cells have been reported to repair osteochondral defects due to exosomal CD73-mediated adenosine activation of AKT and EKT signaling, higher infiltration of CD163+ regenerative M2 macrophages, and a concomitant reduction in the proinflammatory synovial cytokines IL-1*β* and TNF-*α* [16].

In this review, we focus on the relationship between oxidative stress and exosomes and identify extracellular vesicles that are smaller than 220 nm in diameter and carry at least two of the biomarker proteins of exosomes, including CD9, CD81, CD63, TSG101, and syntenin-1 [13], as exosomes according to the study methods and results. We summarize recent studies showing that oxidative stress induced by different inducers affects the yield and components of exosomes and that exosomes derived from various healthy cells or cells under oxidative stress regulate oxidative stress in recipient cells.

## 2. Oxidative Stress Affects the Biogenesis and Release of Exosomes

### 2.1. The Important Regulatory Effects of Oxidative Stress on the Yield and Composition of Exosomes

A low concentration of H_2_O_2_ (5-100 *μ*M) is known to enhance exosomal secretion in lens epithelial cells, HEK293 cells, and leukemia/lymphoma T and B cells [17–19]. The amount of DNA encapsulated in exosomes released from myotubes is enhanced by mild oxidative stress induced using 0.3 mM H_2_O_2_; however, there is no significant change in the yield and size distribution of the exosomes [20].

In addition to H_2_O_2_-induced oxidative stress, other inducers also result in various outcomes, which are summarized in Table 1, in which the outcome of treatment with different types or concentrations of chemicals in several cell types has been shown. Platinum nanoparticles promote the biogenesis and release of exosomes by enhancing the intracellular ROS levels in human lung epithelial adenocarcinoma cancer cells [21]. Oxidative stress induced by mechanical injury increases the exosomal yield in lens epithelial cells [17]. ROS induced by activation of the Ca^2+^-NOX5 (NAD(P)H oxidase 5) (NOX5) signaling axis lead to an increase in exosome release and a simultaneous inhibition of exosome uptake by vascular smooth muscle cells (VSMCs) [22]. Treatment with tert-butyl hydroperoxide (tBHP) induces both oxidative stress and autophagy in human retinal astrocytes (RACs), wherein the size of exosomes enlarges; however, the exosomal yield decreases markedly, without a substantial change in the composition of exosomal content [23]. Atienzar-Aroca et al. showed that oxidative stress induced by 80 mM EtOH increases the release of exosomes encapsulating higher protein and mRNA levels of vascular endothelial growth factor receptors (VEGFRs) from human retinal pigment epithelial cells [24]. However, in their follow-up study, these authors reported that increasing the EtOH concentration beyond 200 mM resulted in no significant effects on the exosomal yield. Notably, different concentrations of EtOH exerted different effects on the level of exosomes containing Bax, Bcl2, and Atg12 [25]. Endogenous ROS induced by homocysteine enhance the release of exosomes containing inflammatory cytokines [26]. Oxidative stress induced by sulforaphane inactivates mammalian target of rapamycin (mTOR) in esophageal squamous cell carcinoma cells, thereby increasing the exosomal yield [27].

### 2.2. Pathways Involving Oxidative Stress That Influence Exosomes

**Figure 1:**
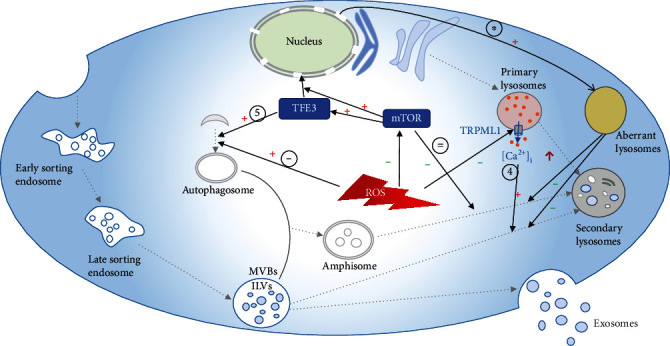
Oxidative stress regulates exosomal yield through different pathways. ① ROS enhance the biogenesis of autophagosomes and promote the degradation of late sorting endosomes [23]. ② ROS inhibit the activity of mTOR, thereby preventing fusion between amphisomes and lysosomes [27]. ③ When mTOR activity is inhibited by ROS, TFE3 is activated and migrates to the nucleus; thus, more nonfunctional lysosomes, which affect the functioning of normal lysosomes, are formed, ultimately causing reduced degradation of MVBs [27]. ④ ROS inhibit the release of calcium ions from lysosomes through TRPML1 and block the fusion between MVBs and lysosomes [26]. ⑤ Several essential proteins that regulate the formation of autophagosomes are upregulated in TFE3-expressing cells [29]. MVBs: multivesicular bodies; ILVs: intraluminal vesicles; ROS: reactive oxygen species; mTOR: mammalian target of rapamycin; TRPML1: transient receptor potential mucolipin 1 channel.

Accumulating evidence has demonstrated that oxidative stress markedly affects exosomal yield by regulating the degradation of multivesicular bodies (MVBs). High oxidative stress promotes the degradation of MVBs by activating autophagy, but low oxidative stress prevents MVBs from being degraded in lysosomes, as shown in Figure 1.

Several recently published studies have indicated that an increase in exosomal yield is associated with inhibition of lysosomal function, which is regulated by oxidative stress in cells [26, 27]. After stimulation with homocysteine (Hcy), endogenous overproduction of ROS in podocytes attenuates lysosomal Ca^2+^ release through the transient receptor potential mucolipin 1 (TRPML1) channel [26]. Inhibition of TRPML1 suppresses Ca^2+^-dependent lysosome trafficking and consequent lysosome-MVB interactions, thereby increasing the secretion of exosomes [26, 28]. TFE3 is a basic helix-loop leucine zipper transcription factor. Dephosphorylated TFE3 migrates from the cytosol to the nucleus and binds to CLEAR elements in the promoters of numerous lysosomal genes to promote their expression and stimulate the biogenesis of lysosomes. Under normal conditions, TFE3 is phosphorylated by activated mTOR and retained in the cytosol by interaction with the cytosolic chaperone 14-3-3 [29]. Zheng and colleagues showed that mTOR is inactivated by ROS, which are increased after treatment with sulforaphane (an isothiocyanate derived from cruciferous vegetables), in esophageal squamous cell carcinoma cells because of turbulence in the GSH/GSSG balance. Consequently, cytosolic TFE3 is dephosphorylated, and more TFE3-mediated abnormal lysosomes, which affect the functioning of normal lysosomes, are formed. As a result, more exosomes are released due to the inhibition of MVB degradation [27].

In addition, the balance between the biogenesis and degradation of autophagosomes, which also directs the degradation of MVBs, is regulated by ROS [23, 25, 27, 29]. Zhu et al. suggested that ROS promote the biogenesis of autophagosomes and that MVBs can be selectively degraded by autophagosomes, further downregulating exosomal release [23]. The proportion of amphisome formation in human retinal pigment epithelial cells increases upon treatment with 600 mM EtOH [25]. Martina et al. found that several essential proteins taking part in the formation of autophagosomes, including ATG16L1 (autophagy-related 16-like 1), ATG9B (autophagy-related protein 9B), GABARAPL1 (GABA type A receptor-associated protein-like), and WIPI1 (phosphoinositide-interacting 1), are upregulated in TFE3-expressing cells [29]. Interestingly, UVRAG (UV radiation resistance-associated gene protein), which plays a role in the degradation of autophagosomes, is upregulated in TFE3-expressing cells at the same time [29]. In addition, autophagic flux is inhibited by ROS through blockade of the fusion between autophagosomes and autolysosomes, thereby increasing the release of exosomes [27].

Furthermore, Benedikter et al. confirmed that the modulation of exosomal release relies on the exofacial GSH level, which is regulated by oxidative stress. Cigarette smoke extract (CSE) causes oxidative stress and enhances the release of exosomes from human bronchial epithelial cells. Furthermore, both H_2_O_2_ and acrolein, which are the oxidative components in CES, significantly affect cell viability; however, only acrolein markedly induces exosomal release, which can be prevented by scavenging thiol-reactive components using NAC or GSH. Acrolein is generated endogenously during inflammation and under oxidative stress. Mechanistically, H_2_O_2_ exerts no substantial effects on cellular GSH levels. However, acrolein increases the intracellular GSH level and concomitantly decreases the exofacial GSH level, which is restored soon after acrolein is washed away from the cells. Notably, the increase in exosomal release is dependent on depletion of the exofacial GSH level [30].

## 3. Oxidative Stress Is Ameliorated by Exosomes Derived from Healthy Cells with Antioxidant Potential

It is well known that some healthy cells, including mesenchymal stem cells (MSCs), neural progenitor cells (NPCs), and astrocytes, resist oxidative damage [31, 32]. However, in clinical settings, their use is limited due to concerns surrounding their safety, ethical issues, or national legislation [10]. Interestingly, their derived exosomes display the same efficacy as the parental cells. Several studies have shown that exosomes derived from MSCs, mouse inner ear stem cells, and human hepatic progenitors prevent oxidative damage both in vivo and in vitro [33–39].

**Figure 2:**
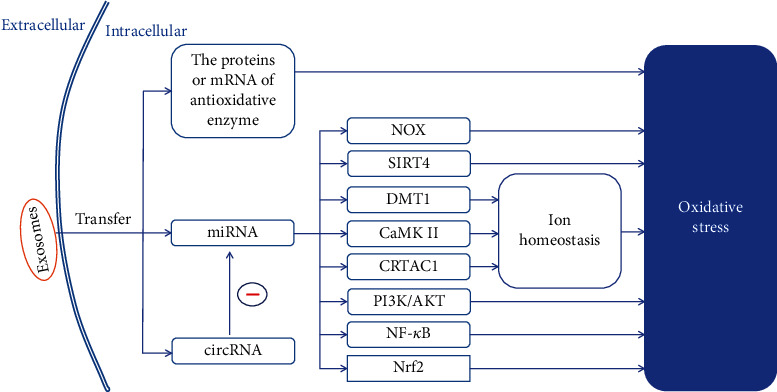
Exosomes derived from healthy cells with antioxidant capacity alleviate oxidative stress in recipient cells through different pathways. Exosomes transfer cellular messages through the delivery of the bioactive molecules enriched in them, thereby alleviating intracellular oxidative stress via different pathways. miRNA: microRNA; NOX: NAD(P)H oxidase; SIRT4: sirtuin 4; DMT1: divalent metal transporter 1; CaMKII: calcium/calmodulin-dependent protein kinase II; CRTAC1: cartilage acid protein 1; PI3K/AKT: phosphoinositide 3-kinase/AKT; NF-*κ*B: nuclear factor-*κ*B; Nrf2: nuclear factor erythroid-2-related factor 2.

Exosomes regulate the intracellular oxidative balance through the delivery of various bioactive molecules, including antioxidative enzymes, miRNAs, and circRNAs in different oxidative stress models (Table 2).  Figure 2 illustrates some pathways through which exosomes regulate intracellular oxidative stress in different cell types.

### 3.1. Exosomes Mitigate Oxidative Stress in Recipient Cells by Directly Delivering Antioxidative Enzyme mRNA or Protein

Several studies have shown that the antioxidant effects of exosomes depend on the encapsulated proteins or mRNAs of antioxidative enzymes. Exosomes derived from human umbilical cord mesenchymal stem cells (huc-MSC-exos) can restore the disturbance in the oxidation and antioxidation balance both in vivo and in vitro. Glutathione peroxidase 1 (GPX1) and manganese-containing superoxide dismutase (MnSOD) are key effector molecules [32, 40]. Notably, huc-MSC-exos present higher MnSOD levels than exosomes secreted from bone marrow (BM-MSC-exos) [32]. After treatment with exosomes secreted from the stem cells of amniotic fluid (AFSC-exos), the expression levels of GSH, superoxide dismutase 1 (SOD1), the antioxidant enzyme thioredoxin reductase 1 (TrxR1), the antioxidant enzyme thioredoxin reductase (TrxR2), and glutathione peroxidase were elevated, thereby leading to a decrease in the ROS level in the neurons of a mouse model of Alzheimer's disease (FAD). The protein level of SOD1 remained stable in AFSC-exos derived from different donors. In addition, the antioxidant efficacy of exosomes is in part related to activation of the PI3K/Akt signaling pathway and concomitant inhibition of NOX4 activity [41]. de Godoy et al. provided evidence that BM-MSC-exos transmit catalase (CAT), thereby completely restoring the basal neuronal ROS level, which was enhanced due to the induction of A*β*Os [31]. BM-MSC-exos exhibit a mitochondrial-protective effect in nucleus pulposus (NP) cells by inhibiting intracellular mitochondrial ROS production upon treatment with H_2_O_2_. The efficacy depends on the mitochondrial proteins transferred from the exosomes to NP cells [42]. Intravenous administration of exosomes secreted from human cardiac resident mesenchymal progenitor cells (CPCs-exos), enriched in SOD, can significantly decrease the ROS levels in the ventricular myocytes of rats treated with doxorubicin alone or along with trastuzumab [43]. Fafian-Labora et al. took advantage of the early passage system of human primary foreskin fibroblasts (HFFF2) derived from 14- to 18-week-old human fetus to obtain a model for young individuals, and the same HFFF2 cells expressing the oncogene H-RAS^G12V^ (iRAS) in a tamoxifen (4OHT)-inducible vector or treated with etoposide were utilized to obtain a model for ageing. Through proteomic analysis, they found that glutathione-s-transferase mu 2 (GSTM2) is highly enriched in exosomes derived from these young cells relative to the ageing model. Exosomes secreted from the young cells reversed the accumulation of ROS in the old donor cells [44].

### 3.2. The Antioxidant Effects of Exosomes Are Dependent on miRNAs

The crucial role of miRNAs encapsulated in exosomes in relieving oxidative stress has been confirmed by many scholars.

miRNAs can relieve oxidative stress by downregulating NAD(P)H oxidase (NOX). Intravenous administration of exosomes secreted from human cardiac resident mesenchymal progenitor cells (CPCs) decreased the level of ROS in the ventricular myocytes of rats treated with doxorubicin alone or along with trastuzumab. Mechanistically, miR-146a-5p delivered from exosomes inhibits the expression of genes that are upregulated by doxorubicin, including Traf6 (the gene encoding tumor necrosis factor receptor-associated factor 6), Smad4 (the gene encoding the signaling effector mothers against decapentaplegic protein 4), Irak1 (the gene encoding interleukin-1 receptor-associated kinase), Nox4 (the gene encoding NAD(P)H oxidase), and Mpo (myeloperoxidase gene) [43]. Administration of exosomes derived from neural progenitor cells elevates the levels of miR-210 and reduces those of NOX2 and ROS through the delivery of miR-210 in a dose-dependent manner in endothelial cells pretreated with angiotensin II (AngII) [45]. Wu and colleagues reported that miR155-5p, which was delivered from exosomes secreted from vascular adventitial fibroblasts of normal rats to vascular smooth muscle cells (VSMCs) of spontaneously hypertensive rats (SHR), downregulates the expression of angiotensin-converting enzyme. Thus, the oxidative damage to the VSMCs, characterized by upregulation of ROS levels, NOX activities, and NOX2 and NOX4 expression, is ameliorated through the ACE (angiotensin-converting enzyme)-Ag II-NOX-ROS axis. Interestingly, NOX4 expression was not restored after treatment with exosomes [46].

miRNA downregulates sirtuin 4 (SIRT4) to mitigate oxidative stress. miR-320a, enriched in exosomes secreted from human amniotic mesenchymal stem cells (hAMSC-exos), reduces the expression of SIRT4 by targeting the 3′ untranslated region of SIRT4 mRNA, thereby decreasing ROS production in a mouse model of premature ovarian insufficiency (POI) and in human primary granulosa cells (hGCs) obtained from POI patients. This is accompanied by a decrease in protein expression levels of adenine nucleotide translocator 2, AMP-dependent kinase, and GTPase optic atrophy type 1, which are downstream target genes of SIRT4 [47].

The beneficial effects of miRNAs in alleviating the effects of oxidative stress rely on the regulation of intracellular ion homeostasis. huc-MSC-exos deliver miR-23a-3p to cardiomyocytes in mice with acute myocardial infarction (AMI) and inhibit the expression of divalent metal transporter 1 (DMT1), thereby increasing GSH levels and decreasing the production of ROS and malondialdehyde (MDA); however, no significant effects on the levels of GPX4 were reported [48]. miR-214 enriched in BM-MSC-exos increases SOD levels and decreases ROS and MDA production by inhibiting the expression of calcium/calmodulin-dependent protein kinase II (CaMK*Π*) in cardiac stem cells treated with H_2_O_2_ [49]. When human lens epithelial cells pretreated with UVB are cocultured with exosomes secreted from adipose-derived stem cells (ASC-exos), the expression of miR10a-5p can effectively downregulate the expression of cartilage acid protein 1 (CRTAC1), thereby decreasing ROS production [50] (Figure 2).

miRNAs can relieve oxidative stress through the PI3K/Akt pathway. Mice that underwent middle cerebral artery occlusion surgery suffered a focal ischemic stroke, which was characterized by ROS overproduction in cerebral microvascular endothelial cells, but the production of ROS was reduced when the mice were administered BM-MSC-exos through their tail vein at the same time. The efficacy of the BM-MSC-exo was dependent on the level of miR-132-3p that was delivered from BM-MSC-exos to cerebral microvessel cells. Furthermore, the effect of BM-MSC-exos enriched with miR-132-3p on decreasing ROS production in H/R-injured ECs was partially abolished by inhibiting the phosphoinositide 3-kinase/AKT/endothelial nitric oxide synthesis (PI3K/AKT/eNOS) signaling pathway in vitro [51].

miRNAs can relieve oxidative stress through the nuclear factor-*κ*B/nuclear factor erythroid-2-related factor 2 (NF-*κ*B/Nrf2) pathway. Exosomes derived from human umbilical cord mesenchymal stem cells (huc-MSC-exos) exerted an antioxidant effect in the hippocampi of seizure-induced mice or H_2_O_2_-stimulated primary cultures of hippocampal neurons through the Nrf2 pathway following the delivery of miR-215-5p, miR-424-5p, miR-31-3P, miR-193b-3p, and miR-200b-3p [10]. miR-29a encapsulated in astrocyte-derived exosomes can restore the expression of miR-29a in recipient cells, thereby alleviating pyroptosis and decreasing apoptosis and oxidative stress in oxygen and glucose deprived (OGD) cells in vitro and in vivo. Liu et al. revealed that miR-29a targets the TP53INP1 (tumor protein 53-induced nuclear protein 1) 3′ UTR and thereby downregulates TP53INP1 expression. As a result, the NF-*κ*B/NLRP3 (nitrogen lipid regulator protein 3) pathway is inactivated, followed by an increase in SOD, GSH-Px, and CAT and a decrease in MDA, apoptosis, inflammatory cytokines (IL-1*β* and IL-18), and pyroptosis levels [52].

### 3.3. Circular RNAs Encapsulated in Exosomes Play a Role in Relieving Oxidative Stress

Circular RNAs (circRNAs), the formation of which involves backsplicing by the canonical spliceosome, are a class of cytoplasmic noncoding RNAs. They are stable and function by interacting with miRNA- or RNA-binding proteins or regulating transcription in cells [53]. Wang et al. demonstrated that circHIPK3 encapsulated in exosomes derived from cardiomyocytes (CMs) can be transferred to cardiac microvascular endothelial cells (CMVECs) to ameliorate oxidative stress, which was characterized by a decrease in ROS and MDA, an increase in SOD expression, and decreased levels of apoptosis. Regarding the mechanism, IGF-1 can inhibit cell apoptosis and induce antioxidant effects. However, miR-29a, which can be abundantly sponged by circHIPK3, is able to target and degrade IGF-1 mRNA in CMVECs. In addition, circHIPK3 was more abundant in exosomes secreted from CMs under hypoxic conditions [54].

## 4. Exosomes Derived from Cancer Cells Regulate Oxidative Stress in Recipient Cells and Promote Metastasis and Drug Resistance

Downregulation of intracellular ROS through the action of exosomes secreted from cancer cells promotes tumor metastasis. Treatment with exosomes secreted from chronic myeloid leukemia cells has been found to decrease ROS production in human primary bone marrow mesenchymal stromal cells and mouse macrophages [55]. Li et al. discovered lower ROS levels, which were positively correlated with increased expression of epidermal growth factor receptor (EGFR), in efficiently metastasizing nasopharyngeal carcinoma (NPC) tissues and highly metastatic NPC cells. EGFR-rich exosomes secreted from highly metastatic NPC cells (H-exos) were internalized by poorly metastatic NPC cells, thereby upregulating intracellular EGFR expression. The upregulated EGFR activated the PI3K/AKT pathway, thereby upregulating ROS production-suppressing genes, including farnesoid X receptor gene and toll-like receptor-4 gene, and downregulating ROS production-promoting genes, including matrix metalloproteinase 2 gene and transforming growth factor *β*1 gene, to decrease ROS production in the poorly metastatic NPC cells, which transformed the poorly metastatic NPC cells into highly metastatic cells after treatment with H-exos. In addition, the authors verified that H-exos can promote low metastatic NPC cell growth and metastasis to the lung and liver via a similar pathway in a xenograft model [56].

Administration of exosomes derived from cancer cells renders chemosensitive cancer cells chemoresistant through downregulation of intracellular ROS levels. Exosomes derived from cisplatin-resistant non-small cell lung carcinoma (NSCLC) tumors are rich in miR-4443, and these exosomes can be absorbed and transfer miR-4443 to cisplatin-sensitive cells. Furthermore, miR-4443 enhances cell viability and decreases the ROS level in cisplatin-sensitive NSCLC tumor cells, thereby promoting cisplatin resistance [57]. Exosomes derived from chemoresistant ovarian cancer (OVCACR-exos) decrease ROS levels in chemosensitive cells and transform them into chemoresistant cells through two major pathways. On the one hand, OVCACR-exos are enriched in plasma gelsolin (pGSN), a multifunctional actin-binding protein. Abundant pGSN can be internalized by chemosensitive cells, leading to Nrf2 overexpression and activation, which increases levels of GSH, an enzyme that eliminates ROS. On the other hand, OVCACR-exos induces apoptosis of CD8+ T cells in the tumor microenvironment (TME). Downregulated IFN-*γ* secreted from CD8+ T cells in the TME decreases the phosphorylation of signal transducer and activator of transcription 1 (STAT1), thereby increasing GSH levels in chemosensitive cells through the Janus kinase/signal transducer and activator of transcription (JAK/STAT) signaling pathway [58]. Administration of exosomes derived from pancreatic cancer (PC) cells pretreated with gemcitabine (Gem-exos) promotes chemoresistance to gemcitabine via two pathways. First, treatment with Gem-exos decreases ROS production by enhancing the expression of superoxide dismutase 2 (SOD2) and CAT, thereby making the recipient cells partially chemoresistant. Second, miR-155 encapsulated in Gem-exos can be delivered to recipient cells and downregulate DCK (a key gemcitabine-metabolizing enzyme) expression. However, other subtypes of extracellular vesicles isolated from the same conditioned medium showed no similar efficacies [59]. In contrast, administration of M1-polarized bone marrow-derived macrophages (M1-BMDMs) markedly increased ROS production in microvascular endothelial cells by activating the NF-*κ*B signaling pathway through the exosomal miR-155/SOCS6/p65 axis. Mechanistically, suppressor of cytokine signaling 6 (SOCS6) can suppress the NF-*κ*B signaling pathway by inducing polyubiquitination and proteasomal degradation of p65. Exosomes secreted from M1-BMDMs (M1-exos) can transfer miR-155, abundantly present in M1-BMDMs, to microvascular endothelial cells and thereby downregulate SOCS6 expression [60].

Although most recent studies have shown that exosomes secreted from cancer cells downregulate ROS levels in recipient cells, Fu et al. suggested that exosomes derived from primary hepatocellular carcinoma can deliver both mRNA and protein of SMAD family member 3 (SMAD3) to circulating tumor cells (CTCs), thereby elevating intracellular ROS, dependent on the overexpression and activation of SMAD3, to facilitate CSC metastasis by promoting cell adhesion [61].

**Figure 3:**
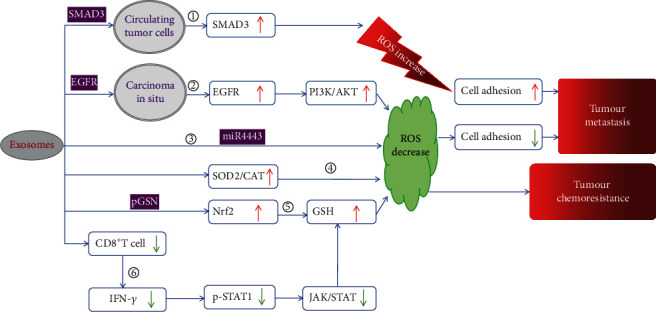
The main mechanisms responsible for the efficacy of exosomes secreted by cancer cells in promoting tumor metastasis and chemoresistance. ① Exosomes secreted from cancer cells deliver SMAD3 mRNA and protein to circulating tumor cells, causing an increase in intracellular ROS due to the upregulation of SMAD3. Finally, the adhesion of circulating tumor cells improves, and circulating tumor cell seeding becomes easier. ② Administration of exosomes derived from cancer cells increases EGFR levels and activates the PI3K/ATK pathway, thereby making cancer cells mobile and able to migrate from the primary carcinoma to the circulatory system due to the downregulation of cell adhesion. ③, ④, ⑤ Exosomes derived from chemoresistant cancer cells decrease ROS levels in recipient cells, making chemosensitive cancer cells chemoresistant through different pathways. ③ Exosomes deliver miR4443 to recipient cells. ④ Exosomes upregulate the expression of SOD2 and CAT in recipient cells. ⑤ Exosomes enhance the expression of Nrf2, followed by upregulation of GSH, thereby benefiting from the delivery of pGSN. ⑥ IFN-*γ* can activate JAK/STAT signaling by phosphorylating STAT1. Exosomes induce CD8+ T cells, which can secrete IFN-*γ*, causing apoptosis in the tumor microenvironment and upregulating GSH via inhibiting of JAK/STAT signaling. ROS: reactive oxygen species; EGFR: epidermal growth factor receptor; SMAD3: SMAD family member 3; PI3K/AKT: phosphoinositide 3-kinase/AKT; SOD2: superoxide dismutase 2; p-STAT1: phosphorylated signal transducer and activator of transcription 1; GSH: glutathione; CAT: catalase; pGSN: plasma gelsolin; Nrf2: nuclear factor erythroid-2-related factor 2; JAT/STAT: Janus kinase/signal transducer and activator of transcription.

The completely distinct efficacies of exosomes derived from cancer cells in regulation of oxidative stress may be attributed to differences in the microenvironment surrounding the recipient cancer cells. Nonetheless, the final efficacy of exosomes secreted from cancer cells is consistent (Figure 3).

## 5. Administration of Exosomes Secreted from Cells under Oxidative Stress Affects the State of Oxidative Stress in Recipient Cells

Several recently published studies indicate that treatment with exosomes derived from cells under oxidative stress leads to oxidative damage. Wang et al. showed that exosomes derived from wounded or H_2_O_2_-pretreated lens epithelial cells, in which ROS levels are increased, can promote the migration of normal lens epithelial cells and change their morphology, which can be inhibited by the ROS inhibitor DPI (diphenyleneiodonium chloride) [17]. Using different cell lines and pancreatic *β* cell-specific miR-15a-/- mice, Kamalden et al. showed that miR-15a is secreted in exosomes specifically by pancreatic *β* cells in vitro and in vivo under high-glucose conditions. Exosomes can be transported to the retina through the circulatory system and transfer miR-15a to retinal cells. Therefore, the level of miR-15a, which is capable of targeting Akt3 to induce oxidative stress, in retinal cells increases [62]. Intravenous injection with exosomes isolated from the sera of rats suffering from hepatic ischemia-reperfusion injury increased the levels of ROS and MDA and reduced the activity of SOD in the hippocampus and cortex of normal rats [63]. Exosomes derived from human uroepithelial cells under oxidative stress induced by ketamine treatment enhanced P38 signaling, thereby activating NF-*κ*B signaling, followed by downregulation of the expression of the antioxidative stress transcription factor—Nrf2 in human uroepithelial cells not treated with ketamine [64]. Nonirradiated naive mice injected with exosomes isolated from 2 Gy irradiated mice through the tail vein were subjected to oxidative damage, and a significant downregulation in the expression of SOD, glutathione-s-transferase (GST), and CAT in the mouse spleen was recorded [65]. Platelet-derived exosomes isolated from the blood of septic individuals with high NADPH oxidase activity induced ROS overproduction in rabbit endothelial cells and rabbit aortic smooth muscle cells in vitro [66].

Surprisingly, cells pretreated with exosomes secreted from other cells under oxidative stress often become resistant to oxidative damage. Fahs et al. suggested that the resistance to oxidative stress in mouse myoblasts could be transferred to recipient cells through exosomes [67]. Eldh et al. reported that MC/9 cells pretreated with exosomes secreted from other MC/9 cells cultured under oxidative stress showed higher resistance to oxidative stress [68]. Lerner et al. showed that exosomes secreted from stressed nonpigmented ciliary epithelium (NPCE-exos), which were induced by 2,20-azobis(2-methylpropionamidine) dihydrochloride (AAPH), can be absorbed by trabecular meshwork cells pretreated with AAPP (OSTM), thereby enhancing their oxidation resistance through several pathways. First, more Nrf2 was presented in the cytoplasm and nucleus of OSTM cells after treatment with NPCE-exos. Second, two key proteins in the Wnt signaling pathway, p-GSK3*β* and *β*-catenin, were downregulated in OSTM cells by treatment with NPCE-exos. Third, several antioxidant genes, including Sod1, Sod2, Gpx1, and Hmox1, were significantly upregulated, and the activities of CAT and SOD were strengthened in OSTM cells after administration of NPCE-exos [69]. Oxidative stress induced by oxygen/glucose deprivation or H_2_O_2_ treatment in astrocytes enhances the secretion of exosomes enriched in the prion protein (PrP). These exosomes deliver PrP to neurons and increase their survival rate under oxidative stress induced by hypoxia or H_2_O_2_ treatment [70]. Exosomes derived from endothelial progenitor cells under different stress conditions display different miR126 expression levels, but their levels are the same as the levels of miR126 in their parental cells. miR126 can be delivered to human brain microvascular endothelial cells in a hypoxia/reoxygenation injury model through exosomes, which negatively regulate ROS production via the PI3K pathway [71].

## 6. Conclusions and Future Directions

In summary, in this review, we discuss the different effects of oxidative stress induced through various stimulating factors on the yield and composition of exosomes and summarize the possible mechanisms proposed in recently published reports. We highlight the beneficial effects of exosomes derived from healthy cells with an antioxidant capacity that relies on the delivery of antioxidative enzymes, miRNAs, and circRNAs. Notably, exosomes secreted from M1-polarized bone marrow-derived macrophages aggravate oxidative stress. Furthermore, exosomes derived from cancer cells can promote metastasis and drug resistance in recipient cancer cells. On the other hand, exosomes secreted from cells under oxidative stress present an entirely distinct effect on the regulation of oxidative stress in recipient cells. The differential effects are attributed to the varied composition of exosomes, as well as the state of the parental cells. Therefore, how oxidative stress impacts exosome composition is of interest.

As an important vehicle for the delivery of cellular messages, exosomes secreted from cancer cells have unfavorable effects on tumor progression and therapeutic responses. Excitingly, oxidative stress can activate autophagy and inhibit the release of exosomes; however, it is also capable of inhibiting the functions of lysosomes, thereby promoting exosome release. More research on the regulatory network of oxidative stress, a crucial intracellular second messenger, is therefore warranted. Inhibition of the release of exosomes from cancer cells to prevent metastasis and chemoresistance under the precise control of intracellular oxidative stress signaling should be examined in future investigations.

## Figures and Tables

**Table 1:** Oxidative stress induced by different inducers in various cells affects the yield and composition of exosomes.

Inducer	Cell type	Exosome yield	Changed exosome composition	Other change	Ref.
5 *μ*M H_2_O_2_	Human lens epithelial cells	Yield increase			[17]
25 *μ*M H_2_O_2_	Human embryonic kidney cells	Yield increase by 28 ± 5%			[18]
50 *μ*M H_2_O_2_	B cell leukemia/lymphoma	Yield increase			[19]
100 *μ*M H_2_O_2_	T cell leukemia	Yield increase			[19]
100 *μ*M H_2_O_2_	Human bronchial epithelial cells	No change			[30]
300 *μ*M H_2_O_2_	Myotube cells	No change	DNA increase		[20]
12 *μ*M acrolein	Human bronchial epithelial cells	Yield increase			[30]
Mechanical injury	Human lens epithelial cells	Yield increase			[17]
30 *μ*M tBHP	Human retinal astrocytes	Yield increase	TSG101, CD81, and CD63 increase; HSP70 reduction	Size increase	[23]
80 mM EtOH	Human retinal pigment epithelium cell	Yield increase	VEGFR protein and mRNA increase		[24]
600 mM EtOH	Human retinal pigment epithelium cell	No change	Bax, Bcl2, and Atg12	Autophagosome increase	[25]
40 *μ*M Hcy	Mouse podocytes	Yield increase		MVB increase	[26]
25 *μ*M sulforaphane	Human esophageal squamous cell carcinoma cells	Yield increase			[27]

**Table 2:** Bioactive molecules transferred via exosomes in different cell or animal models of oxidative stress.

Exosomes	OS^7^ models in vivo/in vitro	Measurement of OS	Bioactivate ingredients	Ref.
Huc-MSC-exos	IRI^2^/H_2_O_2_	SOD^1^, MDA, ROS	MnSOD	[32]
BM-MSC-exos	A*β*Os	ROS	Catalase	[31]
CPCs-exos	Doxorubicin and trastuzumab	ROS	SOD, miR-146a-5p	[43]
Exosomes from young cells	Young cell model	ROS	GSTM2	[44]
Huc-MSC-exos	CCl_4_/CCl_4_ or H_2_O_2_	ROS, 8-OHdG, MDA	GPX1	[40]
BM-MSC-exos	H_2_O_2_	ROS	Mitochondrial proteins	[42]
Huc-MSC-exos	Seizure/H_2_O_2_	CAT, SOD, GSH-Px, FRAP, TOM20, FIS1, COX IV, iNOS, HMGB1, HO-1, Nrf2, 8-OHdG,4-HNE, DT	miR-215-5p, miR-424-5p, miR-31-3P, miR-193b-3p, and miR-200b-3p	[10]
Huc-MSC-exos	AMI	GSH, ROS, MDA	miR-23a-3p	[48]
BM-MSC-exos	IS^5^	ROS	miR-132-3p	[51]
Hamsc-exos	POI/POI	ROS	miR-320a	[47]
BM-MSC-exos	H_2_O_2_	SOD, ROS, MDA	miR-214	[49]
ASC-exos	UVB	ROS	miR-10a-5p	[50]
Exosomes from astrocyte	Oxygen and glucose deprivation	SOD, GSH-Px,CAT, MDA	miR-29a	[52]
M1-exos		ROS	miR-155	[60]
Exosomes from vascular adventitial fibroblasts of normal rats	Primary VSMCs derived from SHR	ROS, NOX activity, NOX2	miR-155-5p	[46]
Exosomes from CMs		ROS, MDA, SOD	circHIPK3	[54]

^1^SOD expression; ^2^ischemia-reperfusion injury; ^3^acute kidney injury; ^4^unilateral ureteral obstruction; ^5^ischemic stroke; ^6^traumatic acute lung injury; ^7^oxidative stress.
